# Energy, exergy, environmental, and economic (4E) analyses of the usability of various nano-sized particles added lubricant in a heat pump system

**DOI:** 10.1016/j.heliyon.2024.e37691

**Published:** 2024-09-13

**Authors:** Gökhan Yıldız, Ali Etem Gürel, Zafer Cingiz, Ümit Ağbulut

**Affiliations:** a Department of Electronics and Automation, Düzce Vocational School, Düzce University, Düzce, 81010, Türkiye; b Department of Electricity and Energy, Düzce Vocational School, Düzce University, Düzce, 81010, Türkiye; c Department of Mechanical Engineering, Mechanical Engineering Faculty, Yildiz Technical University, Istanbul, 34349, Türkiye; d Department of Technical Sciences, Western Caspian University, Baku, Azerbaijan; e Department of Chemistry and Biochemistry, California State University, Los Angeles, State University Drive 5151, 90032, Los Angeles, CA, United States

**Keywords:** Heat pump, Nanolubricant, Thermodynamics, Energy, Environment

## Abstract

The need for energy is rising significantly with the growth of technology in the world. This energy need is largely met by fossil fuels. The enhancement in their prices and the damage they induce to the environment, scientists have turned to alternative energy sources due to the depletion of fossil fuels. In recent years, these alternative energy sources have come to the fore as solar, wind, and wave energy. However, heating and refrigeration systems, whose share of energy consumption in buildings in the world is 40 %, can also compete with these alternative energy sources. In particular, heat pumps (HP) are at a level that can compete with renewable energy sources to seriously reduce this rate. In this study, different nanoparticles were added to the Polyol ester oil (POE) utilized in the compressor to enhance the performance of the HP. Thermodynamic, environmental, and economic performances of the obtained nanolubricants at different concentrations (0.5 wt% and 1 wt%) and flow rates (15, 30, and 45 g/s) were evaluated. The highest COP value of the HP was calculated as 4.14 at 0.5 wt% B-POE at 45 g/s. The best energy consumption in the HP was obtained with 0.5 wt% B-POE nanolubricant with a decrease of 10.96 % at 45 g/s compared to pure POE. The highest exergy efficiency in the HP was calculated at 0.5 wt% B-POE nanolubricant with a 13.53 % increase at 30 g/s compared to pure POE. The best exergoeconomic parameter ($R_{g,ex}$) performance was determined as 3.7148 kWh/$ in 1 wt% TiO_2_-POE nanolubricant at 45 g/s. The best enviro-economic value of 0.16182 ¢/h was obtained with 0.5 wt% B-POE nanolubricant at 45 g/s. In line with the results obtained, it was observed that the B-POE nanolubricant has a performance that can compete with the good-performing TiO_2_-POE nanolubricant.

## 1 Introduction

Fossil fuels have been used for centuries to meet the need for energy, which is one of the basic needs that emerge with the advancement of technological developments in the world day by day. The utilization of alternative energy sources that are more environmentally friendly and do not harm nature has become a necessity today to minimize the negative impacts of fossil fuels [[1], [2]]. Since the utilization of fossil fuels causes global warming and climate change, renewable energy sources such as solar, wind, wave, etc. are the most preferred alternative energy sources [3].

One of these alternative energy sources used today is heat pumps (HP) [4]. At the point of transferring energy, HPs, like other energy transfer and conversion systems, are a system used to transfer existing energy to the desired location. The basic logic of HPs is the process of transferring heat energy from one environment to another environment with less heat energy [5,6]. The fact that the energy used to transport heat energy is much less compared to the energy required to heat the environment during this process, makes HPs stand out among the systems that save on heating [7].

Most of the studies on the development of HPs have been about improving the components of the system, improving the design, and increasing the usability of HPs [8]. In order to HPs to take their place and be preferred among other alternative sources, efforts to increase the performance of the system have increased the energy efficiency significantly [9]. In addition, with the development of nanotechnology in the last decades, there has been an increase in the utilization of nanoparticles in thermal energy systems [10,11]. These nanofluids are generally used to increase performance in thermal systems by adding nanofluids to fluids such as water, oil, etc [12,13].

There are some studies on the utilization of nanofluids in HPs. Shaalan et al. [14] examined the system performance by adding Al_2_O_3_ and CuO nanoparticles into the water used to cool the condenser at various volume concentrations (0.1 %, 0.2 %, and 0.5 %) in a HP used R600a refrigerant. In the experiments, it was determined that when 0.5 % CuO was used instead of pure water to cool the condenser, the COP value was enhanced by 22 % and the energy consumption was reduced by 31 % [14]. Hu et al. [15] conducted experiments on improving the system performance of a ground source HP used in a residence in Wuhan, China, under five different conditions. In the study, in the case of variable flow control with variable pumps, the exergy efficiency of the HP raised to 10.4 %, exergy loss decreased by 51 %, COP value increased to 3.7, and a 60 % reduction in energy consumption was acquired [15]. Shewale et al. [16] obtained nanofluid by adding TiO_2_ and Al_2_O_3_ nanoparticles at different volumetric concentrations (0.1 %, 0.2 %, and 0.3 %) to an HP using R134a and investigated the system performance. In the experiments, an 18 % enhancement in the performance of the system and a 26 % reduction in energy consumption were achieved with the use of 0.3 % Al_2_O_3_ [16]. Li et al. [17] analyzed the effect of 5 % TiO_2_ added nanolubricant by weight on the system performance in a HP using R22. In the experiments, it was determined that the obtained nanolubricant did not change the heat absorbed from the environment in the evaporator and increased the heat discharged from the condenser. A slight decrease in the COP value of the HP was determined in the nanolubricant with TiO_2_ addition [17]. Fedele et al. [18] tested the effects of nanolubricants obtained by adding (0.05 %, 0.1 %, and 0.5 %) TiO_2_ and 0.1 % Single-walled carbon nanotube (SWCNT) at different concentrations by weight to mineral oil (MO) and polyolester (POE) oil in two different compressor oils, on system performance. No impressive enhancement in the system's COP value was determined in the experiments [18]. Mishra et al. [19] examined the effects of nanofluid obtained by adding different volumetric concentrations (0 %, 1 %, 2 %, 3 %, and 4 %) of CuO, Al_2_O_3,_ and SiO_2_ nanoparticles to water in a ground source HP on the system performance. COP value of approximately 3.58 was obtained from 4 % CuO nanofluid compared to the base fluid in experiments [19]. Ahmed et al. [20] examined the effect of the nanofluid obtained by adding Al_2_O_3_ and Cu nanoparticles at different volume concentrations (1 %, 2 %, and 5 %) to water on the system performance. 23 % rise in COP value was observed in 5 % Al_2_O_3_ nanofluid and a 72 % increase in Cu nanofluid [20]. Joshi et al. [21] investigated the performance of POE-Al_2_O_3_ nanolubricant at different concentrations (0.02 wt%-0.1 wt%) to R134a and R600a refrigerants in a vapor compression refrigeration system. An increase of 37.2 % was obtained in POE-Al_2_O_3_ nanolubricant, whose best COP performance was tested with R600a refrigerant at 0.1 wt% concentration. There was a 28.7 % decrease in energy consumption and an 8.9 % decrease in compressor outlet pressure [21]. Jeyakumar et al. [22] tested the performance of nanolubricants obtained by adding different concentrations (0.06 vol%, 0.08 vol% and 0.1 vol%) of Al_2_O_3_, CuO, and ZnO nanoparticles into POE in a vapor compression refrigeration system. Compared to pure POE, an increase of 12.2 % and 3.42 % in the COP value was observed for POE-CuO and POE-Al_2_O_3_ at 0.1 % concentration, respectively. Compared to pure POE, there was a 1.39 % and 0.6 % decrease in energy consumption in POE-CuO and POE-Al_2_O_3_ nanolubricants at 0.1 % concentration [22]. Senthilkumar et al. [23] observed the performance of hybrid nanolubricants at different concentrations (0.2 g/L, 0.4 g/L, and 0.6 g/L) by adding CuO and Al_2_O_3_ nanoparticles into POE in a vapor compression refrigeration system using R600a refrigerant. The COP value increased from 1.17 to 1.6 compared to pure POE. The cooling capacity of the system increased from 160 W to 200 W [23].

HP systems are systems that remain popular with the high-energy efficiency they provide. While nearly 40 % of energy consumption worldwide occurs in buildings, the largest share of this ratio is made up of heating, refrigeration, and ventilation (HVAC) systems. Therefore, improvements that can be made in these systems are of great importance. Due to this circumstance, the utilization of nanofluids in HPs is important. Because, when the literature review is done, it is mentioned that there is a serious performance increase in energy systems using nanofluids. The addition of various concentrations and different types of nanoparticles was examined to notice whether nanofluids are effective on the performance of HP in this study. Therefore, Boron (B) nanoparticle was preferred in addition to TiO_2_ and CuO, which are stated to give good performance in the systems in which it is used in the literature. Since (B) nanoparticles are rarely used in the literature, their performance compared to nanoparticles that are mentioned to be good in studies will be examined. Thermodynamic, economic, and environmental comparisons of nanolubricants added to POE at 0.5 wt% and 1 wt% concentrations and 15, 30, and 45 g/s flow rates were made.

## 2 Material and methods

In this section, the preparation of nanolubricants, the working principle of the experimental setup, and the methods used are explained in detail.

### 2.1 Preparation of nanolubricants

TiO_2_, CuO, and B nanoparticles were utilized with POE to obtain nanolubricants in this study. The nanoparticles utilized in the experiments were obtained from Nanografi Co, Türkiye. Some properties of these nanoparticles are shown in Table 1. This study investigates the suspension of different types of nanoparticles in the base liquid at different weight concentrations.

**Table 1:** Some technical properties of nanoparticles (taken from the supplier).

Specifications	TiO_2_	CuO	B
Purity (%)	+99.5	99.5	+99.55
Density (g/cm^3^)	4.5	6.5	3.58
Average particle size (nm)	45	<77	100
Morphology	Nearly Spherical	Nearly Spherical	Nearly Spherical
Thermal conductivity (W/m.K)	8	33	27

**Fig. 1:**
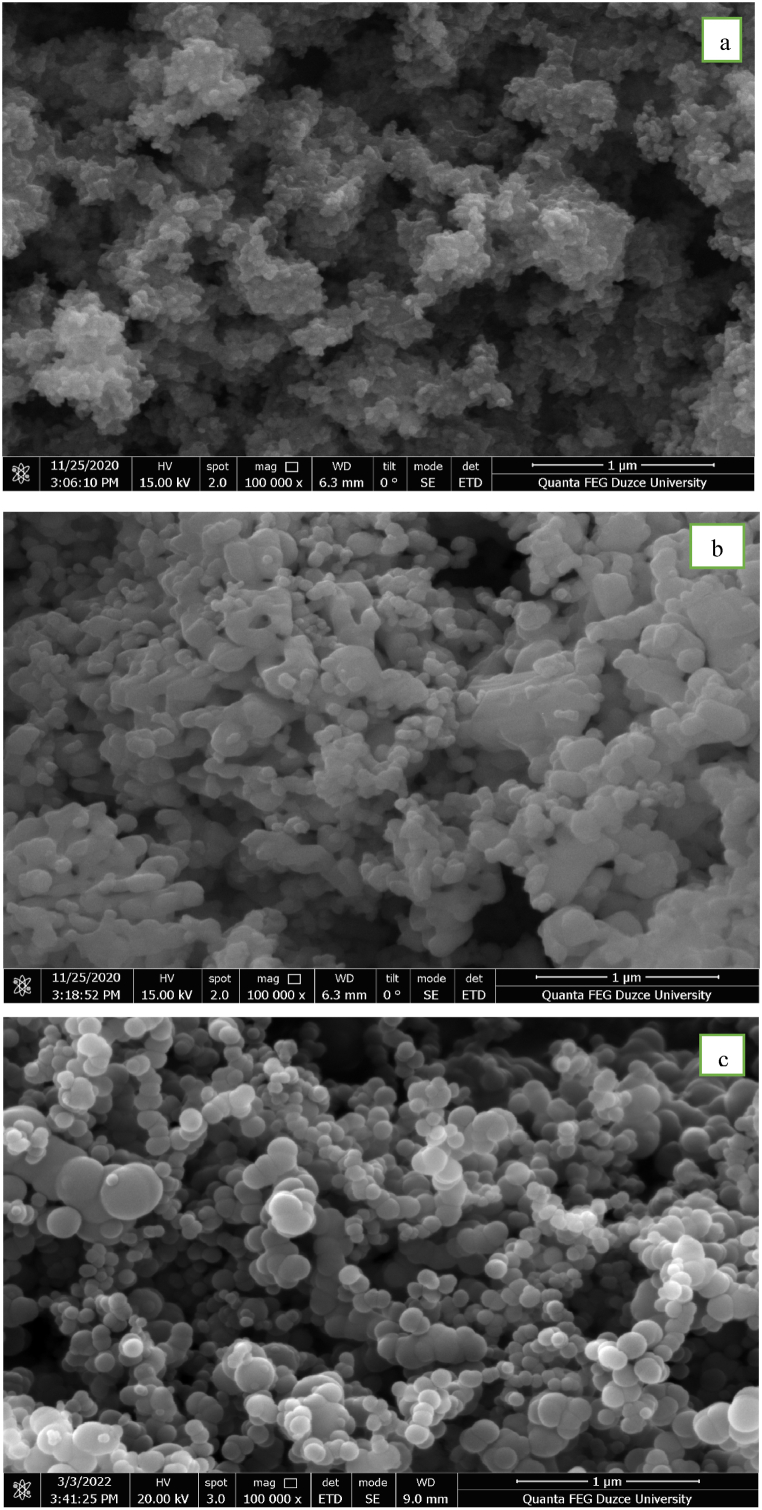
SEM photos of nanoparticles a) TiO_2_, b) CuO, and c) B.

As seen in Table 1, the shape of the nanoparticles used in this study is close to spherical and their sizes vary between 45 and 100 nm. SEM images are given in Fig. 1(a) and (b) and 1 (c) to see the chemical structures of nanoparticles. SEM images were taken at 1 μm size from the FEI brand Quanta FEG 250 model device in the laboratories of Düzce University.

**Fig. 2:**
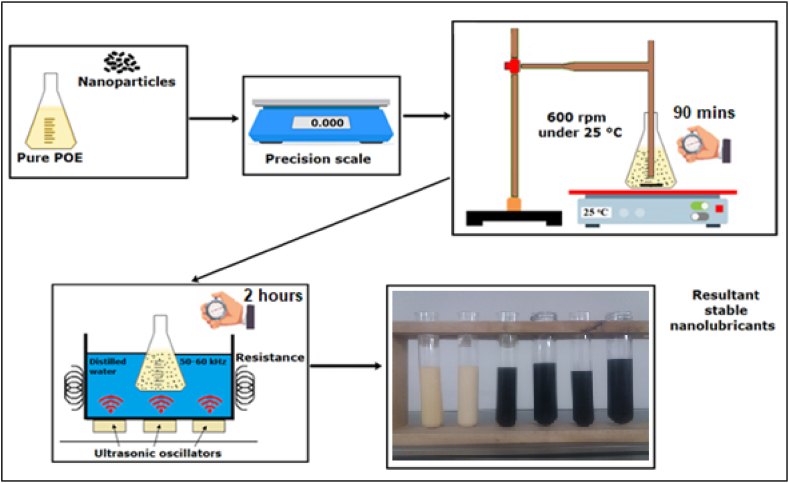
Steps of preparation of different nanolubricants.

In the study, precision balance was utilized to measure the masses of POE and nanoparticles. POE and nanoparticles were blended with the help of a mechanical stirrer at 25 °C for 1 h. After this step, the nanolubricant is subjected to an ultrasonic bath process for 2 h at a frequency of 50 Hz and a power of 250 W under laboratory conditions to ensure homogeneous distribution of the nanoparticles in the POE base fluid. All steps in the preparation of the nanolubricant are shown in Fig. 2.

**Fig. 3:**
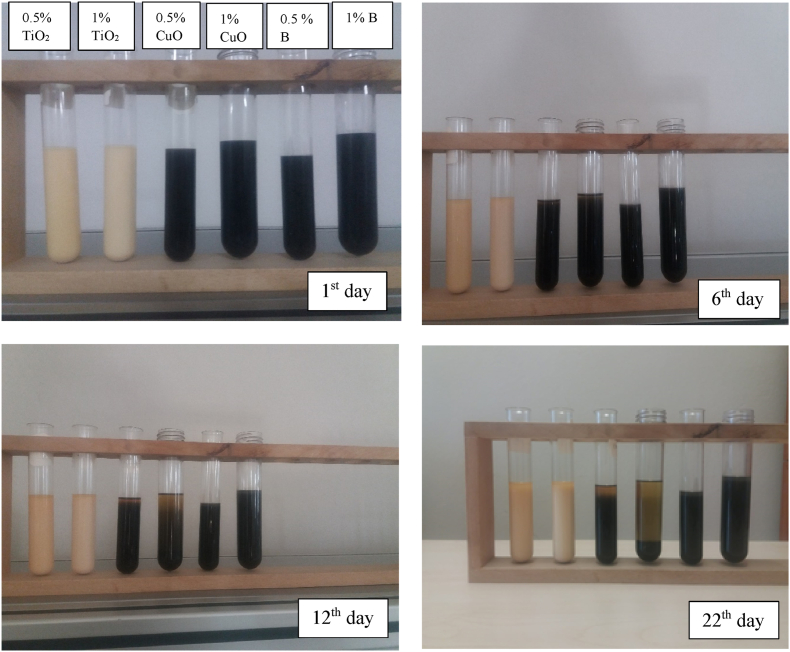
The view of nanolubricant at different periods.

The nanolubricants used in the experiments were prepared at the same time and photographs of the resulting nanolubricants were taken immediately. These taken photographs are indicated in Fig. 3. The color of nanolubricants appears in different colors depending on the color of the nanoparticles as seen in the figure. No sedimentation and/or phase separation was considered in the nanolubricants within 8 h after preparation. After the first 8 h, both phase separation and sedimentation at the bottom began to be observed, albeit slightly. As time passes, the phase separation line begins to move downwards and the amount of nanoparticles accumulated at the bottom increases. Fig. 3 shows the changes of the nanoparticles in the nanolubricant at different times.

Among the nanolubricants, the earliest precipitation was observed in the POE-CuO nanolubricant. This is probably because the CuO nanoparticle begins to precipitate earlier due to its higher density than other nanoparticles. Similar sedimentation events due to the density of the nanoparticle have also been seen in the foregoing studies.

### 2.2 Experimental setup

**Fig. 4:**
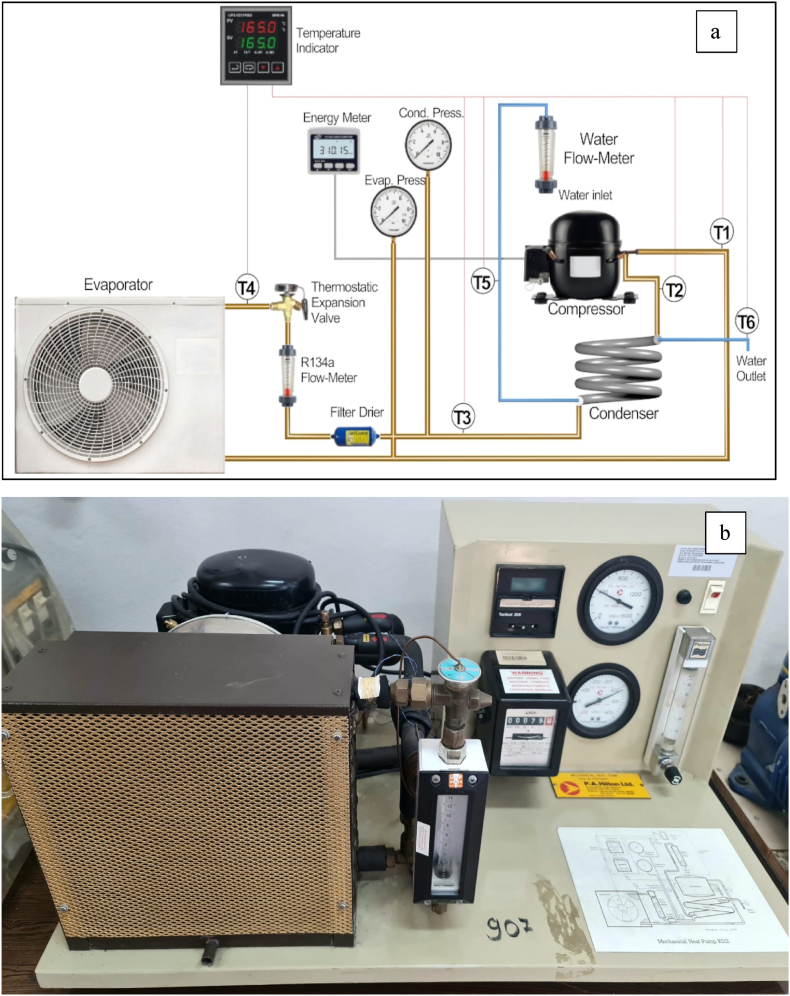
**(**a) Schematic and (b) General view of the HP.

In order to test the final nanolubricant, HP's general and schematic view are shown in Fig. 4(a) and (b). The hermetic piston compressor, thermostatic expansion valve, double-pipe counter-flow condenser, and evaporator make up the HP. Utilizing thermocouples and pressure transmitters respectively, the temperature and pressures within the HP were determined. Flowmeters were utilized to find the R134a and water flow rates in the system.

The low-pressure refrigerant in the HP absorbs heat from the cold air by evaporating in the evaporator. After becoming superheated vapor at the evaporator's outlet, the refrigerant is compressed in the compressor and delivered to the condenser at a higher temperature and pressure. The high-pressure refrigerant in the counter-flow double-pipe condenser transfers its heat to the water that passes through the condenser. The water gets heated and the refrigerant condenses in this manner. After that, the expansion valve lowers the pressure and temperature of the refrigerant before sending it to the evaporator. Thus, the cycle keeps going.

Before beginning the experiments at the HP, the leakage test was conducted. The system was fed N_2_ at 15 bars of pressure in order to accomplish this. For the duration of the leakage test, the system was monitored for 30 min. Then, 170 g of R134a refrigerant and 200 mL of POE were added to the HP from the compressor after it was determined that there was no leak. HP was turned on after charging the POE and refrigerant. The system was maintained for 20 min to achieve equilibrium. Data from the system was then gathered every 30 min at various flow rates (15, 30, and 45 g/s). The same procedures were used to repeat these experiments with the other nanolubricants (POE, 0.5 wt% TiO_2_-POE, 1 wt% TiO_2_-POE, 0.5 wt% CuO-POE, 1 wt% CuO-POE, 0.5 wt% B-POE and 1 wt% B-POE). Relative humidity of 35 % and ambient temperature of 20 °C were observed during the tests. During the nanolubricant replacement, the system's refrigerant was charged and discharged. Refrigerant remains in the system, though, regardless of how much of it is removed from it. For this reason, the system is supplied with N_2,_ and the refrigerant is released before the initiation of each nanolubricant experiment. The refrigerant was charged after this procedure. Table 2 provides specifications for each component in the HP system.

**Table 2:** Technical specifications of HP.

Components	Specifications
Compressor	Displacement: 4.05–9.09 cm^3^
	Cooling capacity: 325–970 W
Evaporator	Capacity: 1 kW
Condenser	Capacity: 1.4 kW
Thermostatic expansion valve	Temperature range: −40/10 °C
	Static superheat: 4 °C
Filter drier	Temperature range: −40/70 °C
	Net volume: 0.464 L

### 2.3 Thermodynamic analysis

In thermal energy systems, thermodynamic analysis is widely used to interpret system performance. Thermodynamic first and second law analyses were applied to analyze the experimental system's performance. These analyses were applied in line with the equations used below:

One of the most important performance indicators for HP systems is COP. The performance of the HP is examined utilizing the following equation. COP is calculated in Equation (1).

(1) $$COP=\frac{{\dot{Q}}_{con}}{{\dot{W}}_{comp,el}}=\frac{\dot{m}c\left(T_{6}-T_{5}\right)}{{\dot{W}}_{comp,el}}$$

This counter-flow double-pipe condenser is thought to operate on the principle of adiabatic heat transfer between the refrigerant and the water. Following is the condenser's energy balance as in Equation (2).

(2) $${\dot{Q}}_{refrigerant}={\dot{Q}}_{water}⟹{\dot{m}}_{refrigerant}.\left(h_{3}-h_{2}\right)={\dot{m}}_{water}.c_{water}.\left(T_{6}-T_{5}\right)$$

The following formula can be used to calculate exergy for unit mass in a flowing system if kinetic and potential energies are disregarded in Equation (3).

(3) $$\psi =h-h_{0}-T_{0}\left(s-s_{0}\right)$$

Equation (4) assumes the following form when the quantity of refrigerant mass flow is multiplied by each point.

(4) $$\dot{Ex}=\dot{m}\left[\left(h-h_{0}\right)-T_{0}\left(s-s_{0}\right)\right]$$

The dead-state conditions in Equation (4) are taken as (T_0_) 25 °C and (P_0_) 101.325 kPa.

Finding the equipment influencing the system's performance requires the spotting of energy destructions in the control volume. The components and the system's overall exergy destruction value offer a way to gauge how successful the improvement is. Finding energy destructions is simple once the exergy balance is established in Equation (5),

(5) $$\sum {\dot{Ex}}_{in}-\sum {\dot{Ex}}_{out}-{\dot{Ex}}_{des}={\left(\frac{dEx}{dt}\right)}_{CV}$$

It can be assumed that there is no energy variation within the control volume concerning time when the control volume is viewed as a continuous system. Thus, the following Equation (6) can be used to determine the exergy destructions for each component.

(6) $${\dot{Ex}}_{des}=\sum {\dot{Ex}}_{in}-\sum {\dot{Ex}}_{out}$$

The following formulas can be used to determine the exergy destructions in the compressor, expansion valve, evaporator, and condenser, respectively, for the exergy analysis of the system under investigation. The general expression of exergy destruction for the condenser is shown in Equation (7).

(7) $$\dot{E}x_{Dest.,Con.}=\left(\dot{E}x_{2}-\dot{E}x_{3}\right)+\left(\dot{E}x_{5}-\dot{E}x_{6}\right)$$

Exergy destruction for the evaporator is calculated in Equation (8).

(8) $$\dot{E}x_{Dest.,Evap.}=\dot{E}x_{4}-\dot{E}x_{1}+{\dot{Q}}_{evap}\left(1-\frac{T_{0}}{T_{evap}}\right)$$

The exergy loss for the compressor is shown in Equation (9). ${\dot{W}}_{Comp,el}$ is calculated as in Equation (10).

(9) $$\dot{E}x_{Dest.,Comp.}=\dot{E}x_{1}-\dot{E}x_{2}+{\dot{W}}_{Comp,el}$$

here;

(10) $${\dot{W}}_{Comp,el}=\frac{{\dot{W}}_{Comp}}{\eta_{el}\times \eta_{mek}}$$

The exergy destruction of the thermostatic expansion valve is calculated in Equation (11).

(11) $$\dot{E}x_{Dest.,\mathrm{Exp}.V.}=\dot{E}x_{3}-\dot{E}x_{4}$$

The following Equation (12) can be used to get the system's total energy destructions.

(12) $$\dot{E}x_{Dest.,Total}=\dot{E}x_{Dest.,Cond}+\dot{E}x_{Dest.,Evap}+\dot{E}x_{Dest.,\mathrm{Exp}.\;V.}+\dot{E}x_{Dest.,Comp.}$$

The HP system's reversible COP is found in Equation (13).

(13) $${COP}_{rev}=\frac{1}{1-\frac{T_{L}}{T_{H}}}$$

Using the following Equation (14), the system's second law efficiency was determined.

(14) $$\eta_{II}=\frac{COP}{{COP}_{rev}}$$

#### 2.3.1 Thermoeconomic analysis

Essentially, being able to compare all systems related to engineering with other systems and making comparisons based on economic data as well as scientific and technical outputs is extremely useful and important in terms of sustainability, which is one of the most important elements of the age. In this sense, it will be possible to increase the value of the work by efficiently evaluating the air-to-water HP system and providing economic benefits. For this purpose, only thermodynamic analysis of energy systems will not be sufficient. Based on this, it is necessary to apply thermoeconomic analysis as well as thermodynamic analysis to an energy system. Making cost calculations of systems and evaluating the findings obtained in line with these calculations is the basic subject of engineering economics. ARC is calculated in Equation (15).

(15) $$ARC=\left(\dot{m}\Delta P/\rho \right)t_{op}CE$$

The capital recovery factor (CRF) is shown in Equation (16). The compound interest rate (i) in the equation is taken as 10 %. The first annual cost (FAC) is calculated in Equation (17). The sinking fund factor (SFF) is shown in Equation (18). n in Equations (16), (17)) refers to the life of the HP. The life of the HP is adopted to be 20 years.

(16) $$CRF=\frac{i{\left(1+i\right)}^{n}}{{\left(1+i\right)}^{n}-1}$$

(17) FAC=CRF×TCI

(18) $$SSF=\frac{i}{{\left(1+i\right)}^{n}-1}$$

The recovery value (ASV) of the HP is calculated according to Equation (19). The salvage value (SV) of the HP is calculated by Equation (20).

(19) ASV=SSF×SV

(20) SV=0.12×TCI

The operating cost of the HP system is taken as 10 % of the initial investment cost [24]. AC is calculated in Equation (21) and $R_{g,ex}$ is calculated in Equation (22).

(21) AnnualCost(AC)=FAC+AMC+ARC−ASV

(22) $$R_{g,ex}=\frac{{\dot{E}x}_{out}}{AC}$$

#### 2.3.2 Thermophysical properties

One of the most significant parameters affecting the system's performance is the thermophysical properties of the fluid used in the system. When determining the thermophysical properties of any fluid, thermal conductivity, and viscosity values generally come to the fore.

Thermal conductivity shows the material's thermal properties in a fluid. Thermal conductivity can be measured with measuring instruments or calculated using models. Maxwell model is considerably utilized to determine the thermal conductivity value in nanofluids. The Maxwell model is generally preferred for spherical nanoparticles. Maxwell's model is defined in Equation (23) [25].

(23) $$\frac{k_{nf}}{k_{bf}}=\frac{k_{np}+\left(n-1\right)k_{bf}-\left(n-1\right)\emptyset \left(k_{bf}-k_{np}\right)}{k_{np}+\left(n-1\right)k_{bf}-\emptyset \left(k_{bf}-k_{np}\right)}$$

One of the factors affecting the nano lubricants performance utilized in the system is dynamic viscosity. Equation (24) was used to calculate the pure POE and nanolubricants’ viscosity utilized [26].

(24) $$\mu_{nf}=\mu_{bf}\;\left(1+2.5\emptyset \right)$$

#### 2.3.3 Environmental analysis

Since the compressors of HP systems operate on electricity, CO_2_ emissions occur in the production of this electrical energy, relying on the source of the power plant. Depending on the compressor consumption, CO_2_ emissions will be calculated for the refrigerant and nanofluid group. It is assumed that the system compressor runs for 18 h to make this calculation. In this study, only compressor power consumption will be assessed. Components's power consumption such as fans will not be taken into account in the analysis. Thus, the daily energy consumption of the compressor able to be calculated simply in Equation (25) (Gürel et al., 2020):

(25) $$W_{comp,el}={\dot{W}}_{comp,el}.t$$

Although there are various approaches to environmental analysis in previous studies, the most commonly utilized evaluation procedure is the calculation of CO_2_ emissions per electricity (kWh). Daily CO_2_ emissions resulting from the compressor's energy consumption ($\varphi_{{CO}_{2}}$) are calculated as shown in Equation (26).

(26) $$\varphi_{{CO}_{2}}=\psi_{{CO}_{2}}.W_{comp,el}$$

In Equation (26), $\psi_{{CO}_{2}}$ is the amount of CO_2_ per kWh resulting from electricity generation in a power plant that uses coal as fuel. When studies in the literature are investigated, it is seen that this value can be taken as 980 gCO_2_/kWh. Considering transmission losses, dispersion losses, and other losses, this value can be taken as around 2 kgCO_2_/kWh [27,28]. The environmental cost analysis value of the HP is given in Equation (27).

(27) $$Z_{{CO}_{2}}=z_{{CO}_{2}}.\varphi_{{CO}_{2}}$$

In the equation, $z_{{CO}_{2}}$ is the international carbon price and varies between $13/tCO_2_ and $16/tCO_2_. This value was taken as 14.5 $/tCO_2_ in the calculations [29,30].

#### 2.3.4 Uncertainty analysis

The total uncertainty of the experimental outcomes is computed in the current paper using Equation (28).

(28) $$W_{R}={\left[{\left(\frac{dR}{dx_{1}}w_{1}\right)}^{2}+{\left(\frac{dR}{dx_{2}}w_{2}\right)}^{2}+\ldots +{\left(\frac{dR}{dx_{n}}w_{n}\right)}^{2}\right]}^{1/2}$$

where $W_{R}$ stands for the experimental results' overall uncertainty (%). The functions $x_{1,}x_{2,}\;x_{3},\ldots .,x_{n}$ are represented by R, which stands for the dimensional shape factor and uncertainty function, respectively. In addition, the uncertainties in the independent variables are denoted by $w_{1,}\;w_{2,}\;w_{3},\ldots .w_{n}$ [31]. The arithmetic average of the three registered data is used to obtain the numerical data shown in the Result and Discussion part of the study. The registered values are repeated 3 times. As a result, the following table lists the primary attributes of every measurement tool utilized in this study. The properties of measurement devices is given in Table 3.

**Table 3:** Specifications of the measurement devices.

No.	Measurement Instrument	Range	Accuracy
1	Flow meter for water	0–50 g/s	1 %
2	Flow meter for refrigerant	0–16 g/s	1 %
3	Thermocouple (K type)	(-30)-130 °C	0.5 %
4	Radwag precision scales	0–220 g	0.001 g
5	Energy meter	0.1–3680 W	0.1 W
6	Pressure transmitter	0–30 bar	1 bar

The uncertainty value is found 1.58 % for COP, 1.87 % for total exergy destruction, and 1.73 % for exergy efficiency.

## 3 Result and Discussions

The system performance because of using different concentrations of nanoparticles in HP is investigated and defined from thermodynamic, environmental, and economic perspectives in this section. Experimental data were taken at 30-min intervals at various flow rates of various concentrations of nanolubricants after the system reached ideal conditions. The experiments are executed in ambient conditions of around 19 °C and 36 % relative humidity.

**Fig. 5:**
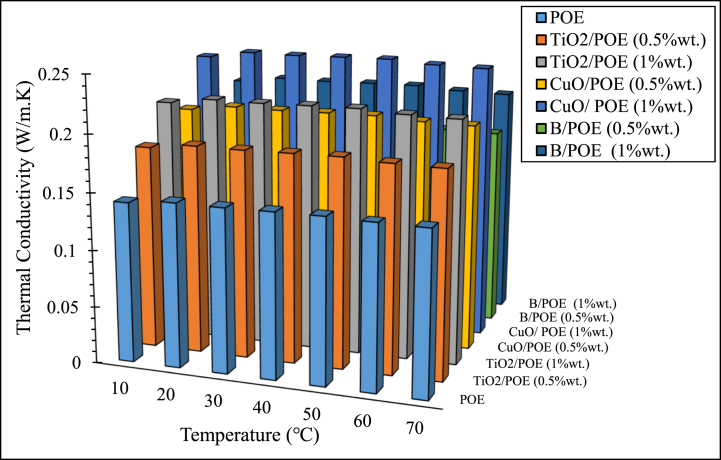
Thermal conductivity changes of nanolubricants in different temperatures.

One of the most significant features affecting performance in thermal energy systems is the lubricant's thermal conductivity utilized in the HP. An enhancement in thermal conductivity is observed by adding nanoparticles to POE. This can be explained by the fact that solid particles have higher thermal conductivity values than liquids, and liquids have higher thermal conductivity values than gases [32,33]. Nanoparticles create a good heat transfer environment by reflecting their high thermal conductivity to the fluid they are added. POE and nanolubricants' thermal conductivity are shown in Fig. 5. Pure POE's thermal conductivity at 70 °C is 0.1434 W/mK. Nanolubricants' thermal conductivity at the same temperature is significantly higher than that of POE. The enhancement in thermal conductivity value due to the nanoparticles' homogeneous dispersion added to the lubricant is defined by increasing the surface-to-volume ratio [34,35]. Due to this circumstance, it is not surprising that the thermal conductivity value of nanolubricants is higher than the thermal conductivity value of POE [36]. It is seen that the thermal conductivity of nanolubricants is higher than POE. This is due to the different sizes of the nanoparticles. Because the surface-to-volume ratio of nanoparticles with smaller grain sizes will be larger [37]. As seen in Fig. 5, the thermal conductivity is enhanced partially with enhancing temperature. Thermal conductivity, which alters according to various temperatures, is similar to previous studies [38,39]. When the temperature of nanolubricants increases, the movements of the molecules in them accelerate [40]. This phenomenon is called Brownian motion. Although this circumstance causes the viscosity to decrease, it causes an increase on average. Higher thermal conductivity is obtained at higher temperatures since this creates a convection impact [41,42]. Nanolubricants' thermal conductivity utilized in HPs is a significant thermophysical feature to obtain higher efficiency from the system.

**Fig. 6:**
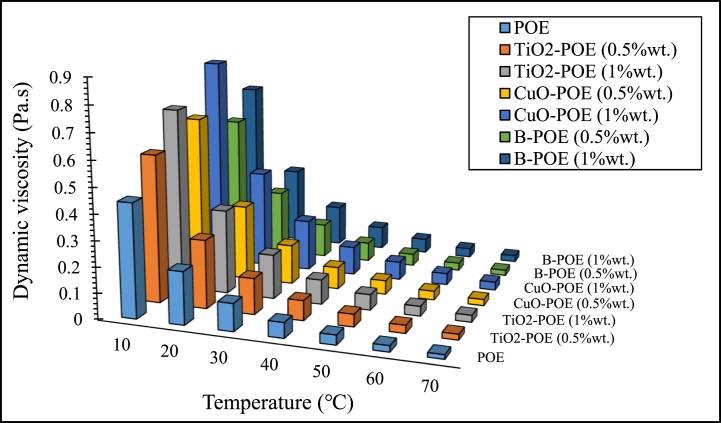
Dynamic viscosity changes of nanolubricants in different temperatures.

One of the significant thermophysical indicators influencing performance in the HP system is viscosity. The friction inside the compressor increases as the viscosity of the fluid utilized in the system increases. The enhancement in friction causes the compressor's temperature to reach high values. For this reason, the change in viscosity of POE and different nanolubricants according to temperature is shown in Fig. 6. It is seen that viscosity values increase by adding different nanoparticles to pure POE. It is observed that POE has the lowest viscosity value when POE and nanolubricants are compared at the same temperature. The reason for the enhancement in viscosity with the adding of nanoparticles can be defined by taking into account the surface area to volume ratio of the fluid to which nanoparticles are added. Hence, the nanolubricants' surface area expands and the resistance to flow increases. Additionally, it is seen that viscosity values in pure POE and nanofluids decrease as the temperature enhances. This circumstance frees the flow by decreasing the cohesive forces between the molecules. Because of this, as the temperature enhances because of reducing cohesive forces, viscosity values in pure POE and nanolubricants reduce. According to Fig. 6, the viscosity of 1 wt% CuO-POE nanolubricant at 10 °C is the highest. The pure POE and nanolubricants' viscosity approach each other as the temperature increases. This phenomenon induces the friction in the compressor to reduce and the compressor's temperature to reduce [43].

**Fig. 7:**
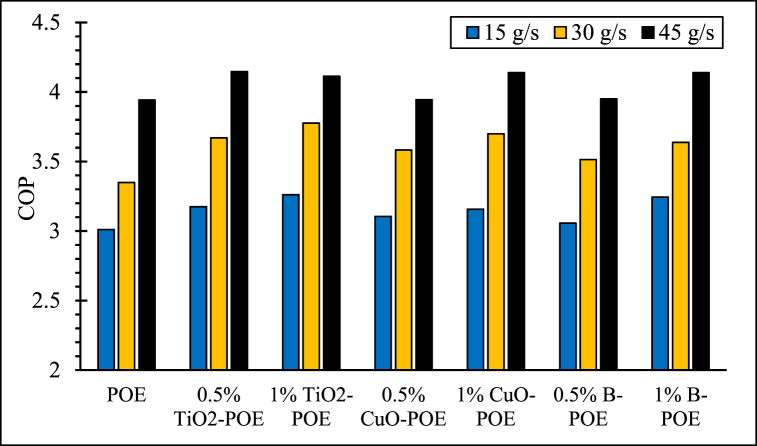
COP changes of HP at various concentrations and flow rates.

Changes in the HP's COPs at various concentrations and flow rates are shown in Fig. 7. HP's COP rises as the flow rate increases as seen figure. This can be explained as more heat is released from the condenser as more water passes through it as the mains water flow rate used to cool the double-pipe nested tube condenser increases. Additionally, the COP value generally increased as the concentrations of nanolubricants used in the HP increased. By increasing the concentration of the nanolubricant used in the compressor, better performance is achieved because of the increase in thermal conductivity ability. In pure POE, COP values were obtained as 3.01, 3.35, and 3.94 at 15, 30, and 45 g/s, respectively. In 0.5 wt% TiO_2_-POE nanolubricant, COP values were obtained as 3.17, 3.67, and 4.15, respectively, at different flow rates. COP values in 1 wt% TiO_2_-POE nanolubricant were determined as 3.26, 3.78, and 4.11, respectively, at different flow rates. In 0.5 wt% CuO-POE nanolubricant, COP values were obtained as 3.11, 3.58, and 3.94, respectively, at various flow rates. COP values in 1 wt% CuO-POE nanolubricant were calculated as 3.16, 3.69, and 4.14, respectively, at various flow rates. COP values of 0.5 wt% B-POE nanolubricant were determined as 3.06, 3.51, and 3.95, respectively, at different flow rates. In 1 wt% B-POE nanolubricant, it was determined as 3.24, 3.64, and 4.14 at different flow rates, respectively. According to the COP obtained, it is seen that nanolubricants at different concentrations perform better than POE. TiO_2_-POE and B-POE nanolubricants at 1 wt% concentration had the best performance at 45 g/s. This shows that the performance of the HP is enhanced with the enhancement in flow rate and concentration [[43], [44], [45]].

**Fig. 8:**
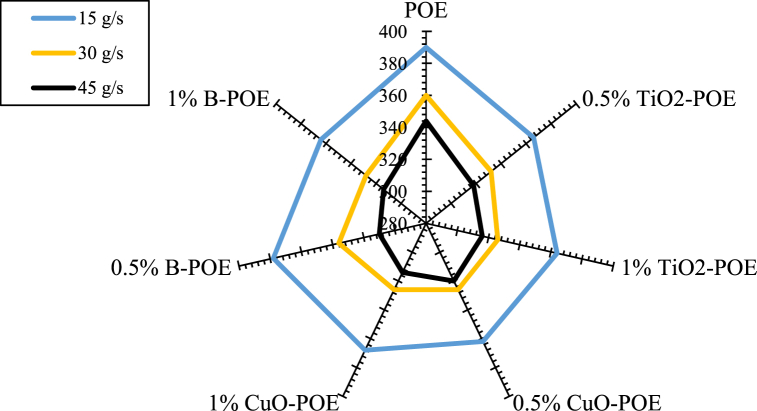
Energy consumption changes of HP at various conditions.

Energy consumption changes of nanolubricants in different conditions are shown in Fig. 8. The HP's energy consumption decreases as seen in the figure as the mains water's mass flow rate used in pure POE and other nanolubricants is enhanced. At the same time, it is seen that as the concentrations of nanolubricants enhance, the energy consumption of the HP decreases significantly. This circumstance causes less friction in the compressor when nanolubricator is used in the HP system. Less power is consumed due to less heating in the compressor. In addition, due to the increase in the performance of the HP with the utilization of nanolubricants, the HP reaches ideal operating conditions faster, and less load is placed on the compressor. In pure POE, energy consumption at 15, 30, and 45 g/s was obtained as 390, 360, and 344 W, respectively. The energy consumption at different flow rates in 0.5 wt% TiO_2_-POE nanolubricator was obtained as 366, 332, and 318 W, respectively. Energy consumption at different flow rates in 1 wt% TiO_2_-POE nanolubricator was determined as 364, 326, and 316 W, respectively. The energy consumption at different flow rates in 0.5 wt% CuO-POE nanolubricant was obtained as 362, 326, and 320 W, respectively. The energy consumption at different flow rates in 1 wt% CuO-POE nanolubricator was calculated as 368, 326, and 314 W, respectively. Energy consumption at different flow rates in 0.5 wt% B-POE nanolubricator was determined as 378, 336, and 310 W, respectively. Energy consumption at different flow rates in 1 wt% B-POE nanolubricator was determined as 364, 328, and 314 W, respectively. According to the COP obtained, it is seen that nanolubricants at different concentrations perform better than POE. TiO_2_-POE and B-POE nanolubricants with 1 wt% concentration had the best performance at 45 g/s. This shows that the performance of the HP is enhanced with the enhancement in flow rate and fraction [45,46].

**Fig. 9:**
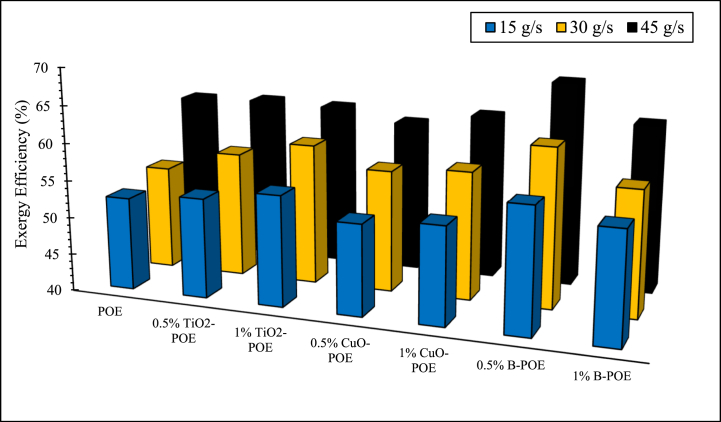
Exergy efficiency changes of HP at various conditions.

POE and nanolubricants' exergy efficiencies at different concentrations in the air-to-water HP are given in Fig. 9. The HP's exergy efficiency is enhanced with the enhancement in mass flow rate. The HP's exergy efficiencies are on average 56 % for pure POE, 57.5 % for 0.5 wt% TiO_2_-POE, 58.67 % for 1 wt% TiO_2_-POE, 56.33 % for 0.5 wt% CuO-POE, 57.20 % for 1 wt% CuO-POE, 61.8 % for 0.5 wt% B-POE, and 58.11 % for 1 wt% B-POE, respectively. While the lowest exergy efficiency was calculated as 52.15 % for 0.5 wt% CuO-POE at 15 g/s, the highest exergy efficiency was obtained as 67.40 % for 0.5 wt% B-POE nanolubricant at 45 g/s. The HP's exergy efficiency is enhanced as the mass flow rate of water passed over the double-pipe counter-flow condenser increases. In general, as the concentrations of nanolubricants enhance with the flow rate, there is a visible enhancement in exergy efficiency. Exergy efficiency data are similar to COP changes as in Fig. 7. The reason for this can be defined by the important role of nanoparticles in heat convection at high flow rate.

**Fig. 10:**
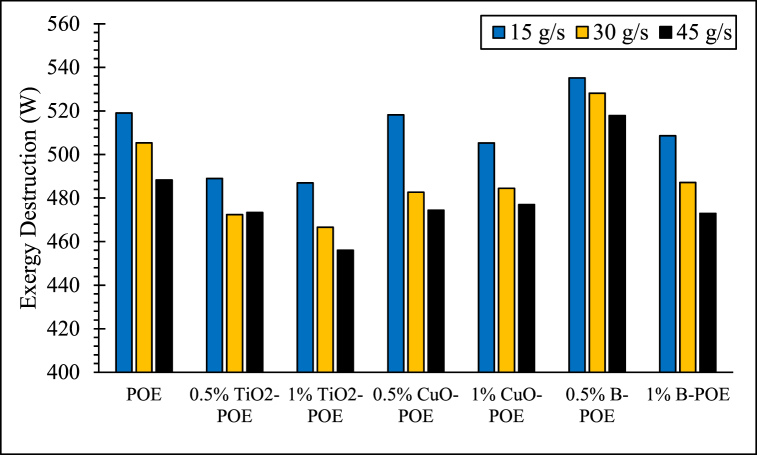
Changes in exergy destruction of the HP at various conditions.

Exergy destruction of POE and nanolubricants at various concentrations and flow rates are shown in Fig. 10. The average exergy destruction of the HP according to different cases is 504.25 W for pure POE, and 478.26 W for 0.5 wt% TiO_2_-POE, 469.86 W for 1 wt% TiO_2_-POE, 491,76 W for 0.5 wt% CuO-POE, 488.93 W for 1 wt% CuO-POE, 527.05 W for 0.5 wt% B-POE and 489.57 W for 1 wt% B-POE, respectively. The lowest exergy destruction is detected in the 1 wt% TiO_2_-POE nanolubricant with 456.04 W and the highest exergy destruction was detected in the 0.5 wt% B-POE nanolubricant with 535.13 W. The HP's exergy destruction varies depending on the various types of nanoparticles used. Additionally, it is seen that the exergy destructions are equivalent to pure POE's COP value and other nanolubricants. POE and nanolubricants with high COP values have lower exergy destruction. This gives exact knowledge about the usability of the HP. With exergy destruction values, the amount of improvement that can be made in the system can be determined and improvements can be made in the system according to these results. In addition, nanolubricants' exergy destruction used in the HP system is lower than pure POE. Nanolubricants increase the performance of the HP and reduce exergy destruction values. Enhancement in the concentrations of nanolubricants reduces exergy destruction [47].

**Fig. 11:**
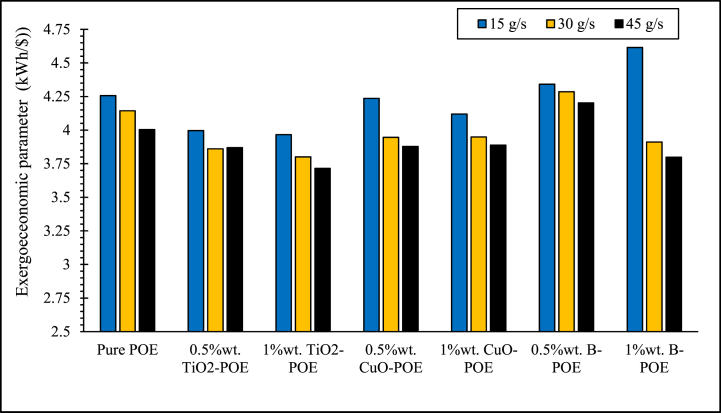
Exergoeconomic changes of nanolubricants at different concentrations and flow rates.

The impacts of using nanolubricants in the HP system on the system performance are mentioned in detail. However, although the enhancement in the performance of HP is a significant indicator, inevitably, this improvement should also be analyzed economically. POE and nanolubricants’ economic changes at various concentrations utilized in HP are shown in Fig. 11. The lowest exergoeconomic parameters are acquired in 1 wt% TiO_2_-POE nanolubricant 3.9668 kWh/$, 3.8008 kWh/$ and 3.7148 kWh/$ at 15, 30 and 45 g/s. The best exergoeconomic performance between pure POE and nanolubricants is observed at 1 wt% TiO_2_-POE. In HPs, energy consumption generally reduces as the flow rate enhances, and accordingly, the economic cost reduces in direct proportion. This can be explained by the reduction in exergy destruction and enhancement in exergy efficiency of the HP with the utilization of nanolubricants.

**Fig. 12:**
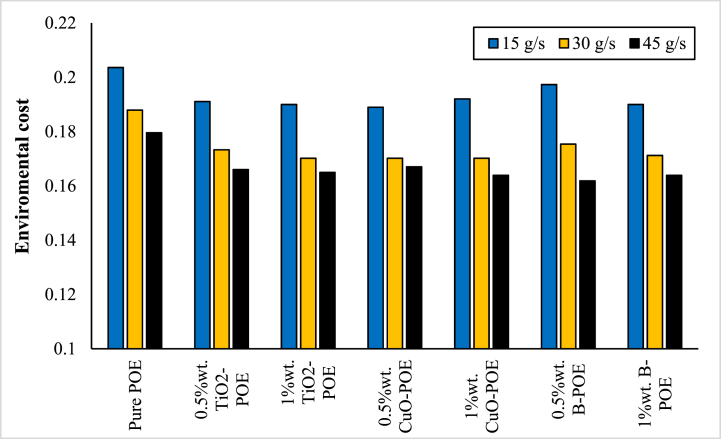
Enviro-economic changes of nanolubricants at different concentrations and flow rates.

In recent years, when a system is analyzed, in addition to its performance and economic evaluation, the environmental effects of the system are also taken into account. Environmental changes of pure POE and nano lubricants at various concentrations and flow rates are shown in Fig. 12. According to the table, as the flow rate of the HP system using pure POE and nanolubricants increases, the environmental cost decreases. The best enviro-economic value of 0.16182 ¢/h was obtained with 0.5 wt% B-POE nanolubricant at 45 g/s.

## 4 Conclusions

In this study, the performance of HP was assessed utilizing TiO_2_, CuO, and B doped nanolubricants with different fractions (0.5 wt% and 1 wt%) utilized at various mass flow rates (15 g/s, 30 g/s, and 45 g/s). Nanolubricants used in HPs were examined from thermodynamic, environmental, and economic aspects. Important notes obtained based on this information are listed below:

✓ Different nanolubricants utilized in the HP importantly increased pure POE's thermal conductivity. The highest thermal conductivity value is detected in 1 wt% CuO-POE nanolubricant, an enhancement of 68.38 % compared to POE. It is observed that thermal conductivity rises importantly with the increase of nanolubricants' concentrations.
✓ An important enhancement in HP performance is observed with the nanolubricants' utilization in the HP. The highest COP increase is 12.54 % at 30 g/s in 1 wt% TiO_2_-POE nanolubricant compared to POE. In terms of performance, the highest COP value was calculated as 4.14 in 1 wt% B-POE nanolubricant at 45 g/s. In a HP, the COP value generally increases as the flow rate and concentration enhance.
✓ Energy consumption in the HP is one of the parameters that directly affects system performance. Additionally, energy consumption affects the payback period when the system is analyzed economically. As the system consumes less energy, the payback period also decreases. It is seen that as the HP's mass flow rate enhances, energy consumption decreases. By using nanolubricants in the HP, energy consumption decreases significantly compared to pure POE. The best energy consumption performance is achieved with 0.5 wt% B-POE, with a decrease of 10.96 % at 45 g/s compared to pure POE.
✓ Nanofluids' thermophysical features play an important role in enhancing system performance. Nanoparticle's grain size is one of the important indicators influencing these thermophysical features. In nanofluids, a decrease in grain size increases the surface area. For these reasons, thermal conductivity and viscosity values rise.
✓ Considering the exergoeconomic analysis of the HP, the $R_{g,ex}$ value decreases parallel to this circumstance, as less energy is consumed as the flow rate enhances. The $R_{g,ex}$ value is partially lower with the use of nanolubricants in the system. Since the cost of B nanoparticle is higher, the $R_{g,ex}$ value is higher than pure POE. The best $R_{g,ex}$ value was obtained in 1 wt% TiO_2_-POE with a decrease of 8.21 % at 45 g/s compared to pure POE.
✓ When nanolubricants are used at different flow rates, HP's total exergy destruction changes. Exergy destructions show the HP system's usability. It gives an idea about where improvements should be made in the system. The total exergy destruction of whole nanolubricants reduces as the mass flow rate enhances. Total exergy destruction is inversely proportional to COP values. The HP's total exergy destruction decreases as the HP's COP value increases.
✓ The best performance was seized at 0.5 wt% B-POE at 30 g/s with an increase of 13.53 %, when the exergy efficiency of nanolubricants was compared to pure POE. Exergy efficiency generally increased with increasing concentration.

The utilization of nanofluids to increase performance in thermal energy systems is completely high when the literature is examined. However, this orientation is quite low in HP systems. One of the important aims of this study is to show that nanofluids used in refrigeration systems or solar energy systems also provide a significant improvement in HP systems. For this purpose, various nanoparticles at different concentrations were added to the POE utilized in the air-to-water HP to obtain a nanolubricant, and the system performance was evaluated. As a novelty in this study, B nanoparticle, which is rarely used in thermal energy systems, was used, unlike nanoparticles that are used extensively in studies. B-POE nanolubricant performed well sufficient to compete with TiO_2_-POE nanolubricant. This circumstance may have a serious benefit for Türkiye, which has 80 % of the world's boron reserves.

Nanotechnology has paved the way for nanolubricants to replace fluids traditionally used in heat pump and refrigeration systems. The use of these fluids has become possible thanks to significant developments in nanotechnology. The use of these nanolubricants has resulted in increased thermal efficiency of the system, energy saving, and improved dissolution and heat transfer ability. While nanolubricants have positive aspects in the system, problems may also occur that may have negative effects on the functioning of the system.

One of the serious challenges when preparing nanolubricants is ensuring dispersion stability within the nanoparticles. Another problem is temperature fluctuations, particle aggregation, and long-term degradation of mixtures obtained with surfactants. The lubrication mechanism of nanoparticles is complex and further research is required to obtain nanolubricants that do not contain harmful substances. Increased viscosity causes adverse effects on refrigeration and heat pump systems. The effect of viscosity must be examined in detail to minimize the pumping power associated with the pressure drop in the compressor. Additionally, oil separators used in refrigeration and heat pumps are installed to isolate nanoparticles within the compressor and minimize their accumulation effects on the mechanical parts of the system. Therefore, the possibility of nanoparticles remaining in the refrigeration system increases. Therefore, to understand the importance of nanolubricants in the compressor, future studies can examine the effectiveness of oil separators in effectively isolating nanoparticles. The optimum concentration of nanolubricants used in refrigeration and heat pumps has still not been determined in general. The optimum concentration of nanolubricants can be determined in future studies. It is known that nanolubricants cause short-term performance increases in heat pump and refrigeration systems. However, how nanolubricants will perform on the system in the long term can be analyzed.

## Data and code availability statement

No data was used for the research described in the article.

## CRediT authorship contribution statement

**Gökhan Yıldız:** Writing – original draft, Methodology, Investigation, Data curation, Conceptualization. **Ali Etem Gürel:** Writing – review & editing, Writing – original draft, Supervision, Methodology, Conceptualization. **Zafer Cingiz:** Writing – review & editing, Writing – original draft, Resources, Conceptualization. **Ümit Ağbulut:** Writing – review & editing, Writing – original draft, Methodology, Conceptualization.

## Declaration of competing interest

The authors declare the following financial interests/personal relationships which may be considered as potential competing interests: The author ÜA serves as an AE in Heliyon.
